# The epigenetic underpinnings of lower back pain

**DOI:** 10.1002/ctm2.868

**Published:** 2022-06-08

**Authors:** Richard L. Bennett

**Affiliations:** ^1^ Department of Medicine, Division of Hematology and Oncology University of Florida Gainesville Florida USA

1

Lower back pain is a leading cause of disability in western countries, and about 20% of those affected by lower back pain develop a chronic condition.^1^ In addition to the personal impact, the loss of productivity associated with chronic lower back pain causes a large socioeconomic burden for western society. Chronic lower back pain is caused by intervertebral disc degeneration (IVDD) characterized by the breakdown of one or more of the discs that separate the spinal vertebrae. Current treatments for IVDD focus on relieving the symptoms of back pain but often do not address the underlying molecular mechanisms of disc degeneration. Therefore, an important focus of current research is to identify the various factors responsible for disc degeneration, as well as strategies for regeneration.

Intervertebral discs are multi‐component structural tissues composed of an inner hydrated gel‐like nucleus pulposus (NP) surrounded by an outer fibrous region and a cartilaginous endplate, which together normally provide cushioning between vertebrae and absorb pressure put on the spine. Under compressive loads, the NP distributes hydraulic pressure within each disc in all directions. The NP consists of collagen fibrils, proteoglycan aggrecans, and NP cells (NPCs). NPCs reside in an environment with limited vasculature and generate energy through anaerobic glycolysis. These cells synthesize collagen and secrete chemokines, matrix proteases, cytokines, and growth factors.^2^ The pathophysiology of IVDD is not fully understood; however, environmental and genetic factors are reported to contribute to NPC senescence that leads to increased catabolism of the extracellular matrix, loss of proteoglycan and water content in the NP, and failure of the intervertebral disc biomechanical properties.

A recent report from Li et al. reveals a new mechanism for how altered epigenetic mechanisms contribute to NPC senescence and IVDD (Figure 1).^3^ Epigenetic mechanisms regulate heritable gene expression patterns without affecting the underlying DNA sequence. Decades of research suggests these mechanisms are responsible, at least in part, for the aging process. Age related variations of epigenetic mechanisms such as DNA methylation, noncoding RNAs, chromatin remodeling and histone posttranslational modification produce transcriptional changes responsible for many aging‐related diseases such as cancer and heart failure. By comparing gene expression from NPCs isolated from normal or degenerated discs, the authors found that IVDD was associated with a gene expression signature of NPC senescence. Significantly, the expression of the ALKBH5 gene, a *N* ^6^‐methyladenosine (m^6^A) demethylase, was found to be enriched in senescent NPCs and expression of senescent markers was determined to be dependent on ALKBH5 expression and activity. The authors determined that the histone lysine demethylase KDM4A was increased in senescent NPCs and bound to the promoter region of the ALKBH5 gene, causing a decrease in histone H3 lysine 9 trimethylation at the ALKBH5 promoter, which promoted increased ALKBH5 expression. In addition, the authors found that the DNMT3B mRNA half‐life was shorter in ALKBH5 knockdown cells. The m^6^A peak of DNMT3B was enriched in the 3′UTR region of DNMT3B mRNA, and by RNA pull down it was revealed that YTHDF2 bound to this m^6^A site in the DNMT3B mRNA. In both normal and senescent NPCs, YTHDF2 inhibition increased the stability and expression of DNMT3B mRNA. These results suggest that in senescent NPCs increased ALKBH5 results in decreased YTHDF2‐mediated decay of the DNMT3B transcript.

**FIGURE 1:**
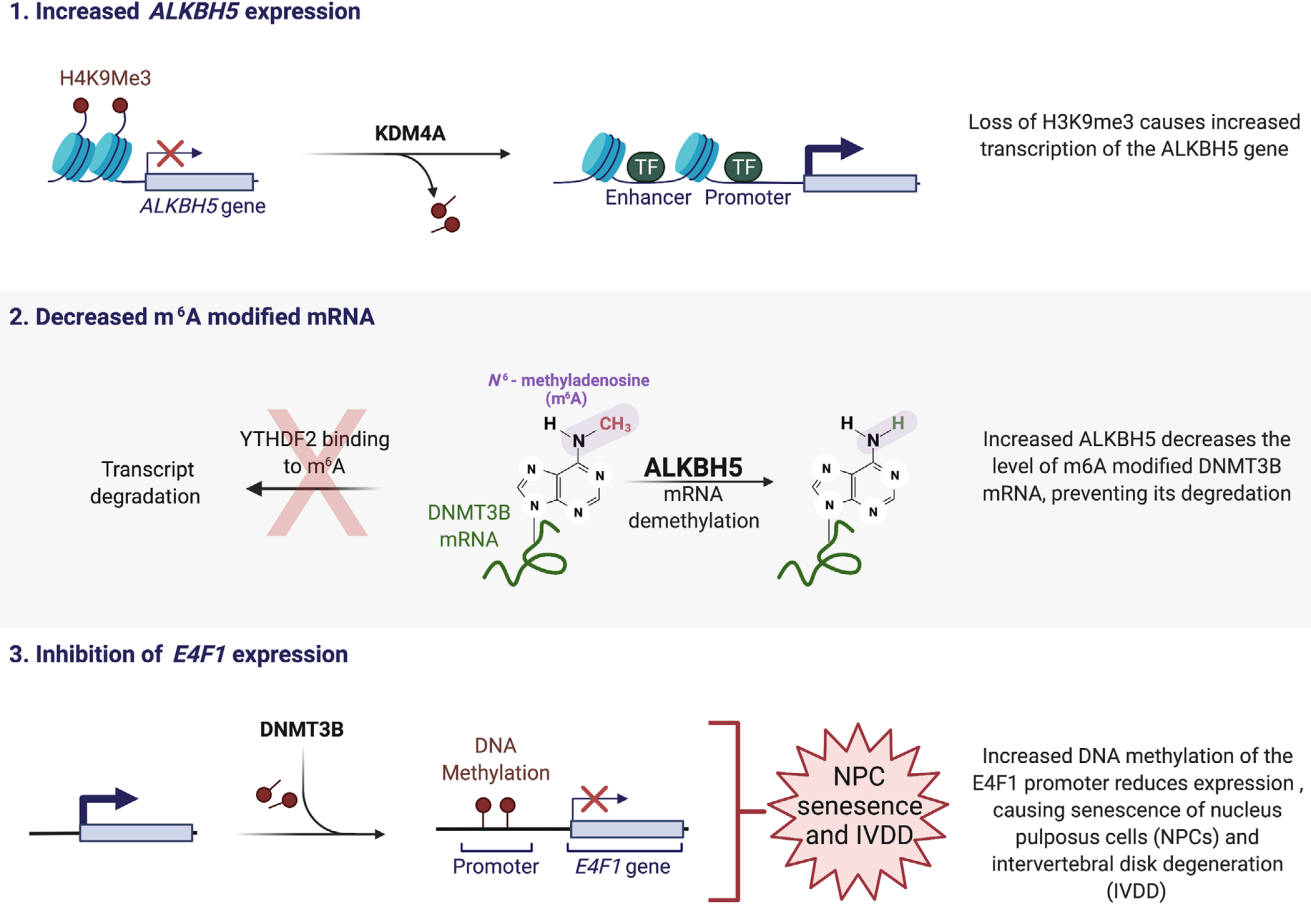
Epigenetic mechanism of intervertebral disc degeneration. 1. In senescent nucleus pulposus cells (NPCs) expression of KDM4A is increased, which decreases the level of histone H3 lysine 9 trimethylation (H3K9me3) at the ALKBH5 promoter to enable gene expression. 2. Increased ALKBH5, an *N* ^6^‐methyladenosine (m^6^A) demethylase, decreases the amount of m^6^A found on the 3′UTR of DNMT3B mRNA, preventing YTHD2‐dependent mRNA degradation. 3. Increased DNMT3B causes increased methylation of the E4F1 promoter which inhibits E4F1 expression. Loss of E4F1 leads to NPC senescence and disc degeneration

To further characterize the role of DNMT3B in NPC senescence and IVDD the authors knocked down DNMT3B expression in NPCs. Decreased DNMT3B blocked tumor necrosis factor‐alpha (TNFα)‐induced senescent of NPCs. In a rat model, knockdown of DNMT3B decreased IVDD and the aging process of NPCs by MRI imaging and histological analysis. Since the authors had found that E4F1 was the only gene associated with both cell cycle and senescence whose expression was decreased in senescent NPCs, they examined the methylation status of the E4F1 promoter in NPCs. The results showed that senescent NPCs from degenerated individuals had increased DNA methylation at the E4F1 promoter compared with normal NPCs. Inhibition of DNMT3B could partially abolish E4F1 promoter methylation and reestablish E4F1 expression. Significantly, when E4F1 expression was decreased in NPCs by siRNA, these cells became senescent while overexpression of E4F1 in NPCs could reverse TNFα‐induced senescence, and enforced expression of E4F1 could partially abolish the pro‐senescence effects of ALKBH5 and DNMT3B. These results reveal a critical role in the m^6^A‐modification of DNMT3B mRNA in NPC senescence and IVDD.

The author's findings reveal a new epigenetic underpinning of NPC senescence and IVDD. Importantly, since epigenetic mechanisms are reversible these results suggest new therapeutic strategies for IVDD that target ALKBH5, and DNMT3B to reactivate E4F1 expression. Future work must now be directed to better characterize other genes altered by the m^6^A imbalance as well as more global analysis of increased DNA methylation in senescent NPCs. Increased ALKBH5 was attributed to increased KDM4A in senescent NPCs, but what other genes are affected by increased KDM4A and how is the level of KDM4A unbalanced in senescent NPCs? The multifunctional protein E4F1 controls the balance between proliferation and cell survival through multiple diverse mechanisms such as the p53 and pRB tumor suppressors, the CDK inhibitor p21, and the transcriptional repression of the cyclin A2 promoter, and the regulation of the DNA‐damage response. It will be important to determine which of the functions of E4F1 may be directing the senescence of NPCs. By improving our understanding of the molecular mechanisms that drive IVDD, we will be able to develop more effective back pain therapies in the future.

## CONFLICT OF INTEREST

The author declares no conflict of interest.

## References

[1] Dutmer AL , Schiphorst Preuper HR , Soer R , et al. Personal and societal impact of low back pain: the Groningen spine cohort. Spine. 2019;44(24):E1443‐E51. 31369481 10.1097/BRS.0000000000003174

[2] Sampara P , Banala RR , Vemuri SK , Av GR , Gpv S . Understanding the molecular biology of intervertebral disc degeneration and potential gene therapy strategies for regeneration: a review. Gene Ther. 2018;25(2):67‐82. 29567950 10.1038/s41434-018-0004-0

[3] Li G , Luo R , Zhang W , et al. m6A hypomethylation of DNMT3B regulated by ALKBH5 promotes intervertebral disc degeneration via E4F1 deficiency. Clin Transl Med. 2022;12(3):e765. 35340126 10.1002/ctm2.765 PMC8957938

